# Which biomarkers are predictive specifically for cardiovascular or for non-cardiovascular mortality in men? Evidence from the Caerphilly Prospective Study (CaPS)

**DOI:** 10.1016/j.ijcard.2015.07.106

**Published:** 2015-12-15

**Authors:** Christopher C. Patterson, Stefan Blankenberg, Yoav Ben-Shlomo, Luke Heslop, Antony Bayer, Gordon Lowe, Tanja Zeller, John Gallacher, Ian Young, John Yarnell

**Affiliations:** a Centre for Public Health, Queen's University Belfast, Belfast, UK; b University Heart Centre Hamburg, Clinic for General and Interventional Cardiology, Hamburg, Germany; c Social and Community Medicine, University of Bristol, UK; d Institute of Primary Care & Public Health, Cardiff University School of Medicine, Cardiff, UK; e Institute of Cardiovascular and Medical Sciences, University of Glasgow, Glasgow, UK; f Department of Psychiatry, Warneford Hospital, Oxford, UK

**Keywords:** Biomarkers, Cardiovascular mortality, Non-cardiovascular mortality, Epidemiology

## Abstract

**Objective:**

To examine a panel of 28 biomarkers for prediction of cardiovascular disease (CVD) and non-CVD mortality in a population-based cohort of men.

**Methods:**

Starting in 1979, middle-aged men in Caerphilly underwent detailed medical examination. Subsequently 2171 men were re-examined during 1989–1993, and fasting blood samples obtained from 1911 men (88%). Fibrinogen, viscosity and white cell count (WCC), routine biochemistry tests and lipids were analysed using fresh samples. Stored aliquots were later analysed for novel biomarkers. Statistical analysis of CVD and non-CVD mortality follow-up used competing risk Cox regression models with biomarkers in thirds tested at the 1% significance level after covariate adjustment.

**Results:**

During an average of 15.4 years follow-up, troponin (subhazard ratio per third 1.71, 95% CI 1.46–1.99) and B-natriuretic peptide (BNP) (subhazard ratio per third 1.54, 95% CI 1.34–1.78) showed strong trends with CVD death but not with non-CVD death. WCC and fibrinogen showed similar weaker findings. Plasma viscosity, growth differentiation factor 15 (GDF-15) and interleukin-6 (IL-6) were associated positively with both CVD death and non-CVD death while total cholesterol was associated positively with CVD death but negatively with non-CVD death. C-reactive protein (C-RP), alkaline phosphatase, gamma-glutamyltransferase (GGT), retinol binding protein 4 (RBP-4) and vitamin B6 were significantly associated only with non-CVD death, the last two negatively. Troponin, BNP and IL-6 showed evidence of diminishing associations with CVD mortality through follow-up.

**Conclusion:**

Biomarkers for cardiac necrosis were strong, specific predictors of CVD mortality while many inflammatory markers were equally predictive of non-CVD mortality.

## 1 Introduction

There is increasing evidence that coagulation and inflammatory factors play an important role in the pathogenesis of cardiovascular diseases (CVD), and this appears to be largely initiated and progressed by atherothrombotic mechanisms [1]. Coagulation factors, which include key markers of individual components of the coagulation cascade and inflammatory markers, have been extensively investigated in many cohort studies, which included the Caerphilly Prospective Study (CaPS) [2], in relation to acute coronary and stroke outcomes. Eighteen inflammatory and haemostatic markers [3] have previously been investigated in an earlier phase of this study.

Other pathways have also been linked with risk of CVD. These include the following: markers of *cardiac necrosis* (troponins) and *vascular function* (natriuretic peptides) [4], which are routinely used in clinical diagnostic practice; *markers of the acute phase response* (to infection), for example fibrinogen, viscosity and WCC [5], C-reactive protein [6],and IL-6 [3]; *markers of leukocyte activation*, pregnancy associated protein A (PAPP-A) [7,8], and growth differentiation factor (GDF-15) [9,10]; *markers of endothelial function*, such as the cellular adhesion molecules, which are expressed on the surface of vascular endothelial cells, e.g. vascular cell adhesion molecule-1 (VCAM-1) and endothelial-leucocyte adhesion molecule-1 (E-selectin) [11,12]; *markers of insulin resistance* e.g. glucose, retinol binding protein 4 (RBP-4) [13,14], and fetuin-A [15]; a *marker of renal function*, cystatin-C [16,17]; and pyridoxine, vitamin B_6_ [18,19], which has a role in the metabolism of homocysteine. *Liver enzymes*, such as gamma-glutamyltransferase (GGT), have also been shown to be independently associated with the risk of CVD in the general population [20,21].

Few studies have investigated the role of CVD biomarkers in the prediction of non-CVD mortality, but recently the Atherosclerosis Risk in the Communities (ARIC) study reported that troponin, NT-pro-BNP and C-reactive protein were associated with risk of CVD, and also some non-CVD causes [22]. In this report we examine whether a panel of biomarkers predicts CVD or non-CVD mortality in a long-term follow-up of men recruited from the general population of the town of Caerphilly in Wales, UK.

## 2 Methods

### 2.1 Study population

CaPS was established in 1979 when men aged 45 to 59 years from the general population of this South Wales town and its surrounding villages were recruited; 89% of the eligible population was examined and the cohort was re-examined at approximately 5 yearly intervals. Men were invited to attend an afternoon or evening clinic at which informed consent and a detailed medical and lifestyle history were obtained, the London School of Hygiene and Tropical Medicine (LSHTM) chest pain questionnaire was administered, a full 12-lead electrocardiogram (ECG) was recorded, and weight and blood pressure were measured. This report is based on the second follow-up examination of the men (phase 3) in the period 1989–1993 when the men were predominantly aged 55–69 years. 2171 men were examined, and a fasting blood sample was obtained subsequently from 1911 men (88%). All men gave written informed consent. This project was approved according to the guidelines of the UK National Research Ethics Service by the South East Wales Research Ethics Committee in July 2009 (reference 09/WSE04/32).

### 2.2 Blood collection, storage, and analysis

At a separate appointment, shortly after the clinical examination, a venous blood sample was collected from each man after an overnight fast. Fresh samples were transported to Frenchay Hospital, Bristol for measurement of fibrinogen, viscosity and WCC as described previously [5]. Similarly routine biochemistry tests and lipids were analysed at the University Hospital of Wales, Cardiff. Other aliquots were taken and plasma or serum were separated within one hour and stored at − 20 °C for up to 6 h and then at − 70 °C until laboratory analysis in 2010–13.

### 2.3 Biomarker assays

Assays for high-sensitivity troponin, B-type natriuretic peptide, C-reactive protein, GDF-15, cystatin-C, creatinine, ferritin and lipoprotein-a (Lp-a) were performed in the Mainz Biomarker laboratory as described elsewhere [23]. VCAM-1, E-selectin and IL-6 were assayed in Glasgow using commercial enzyme-linked immunosorbent assay (ELISA) kits (R&D Systems, Abingdon, UK). PAPP-A (Demeditec Diagnostics, Kiel, Germany) RBP-4 (R&D Systems, Abingdon, UK), fetuin-A (BioVendor Laboratorni Medicina, Brno, Czech Republic), and IL-6 receptor (R&D Systems, Abingdon, UK) were assayed in Belfast using commercial ELISAs, and vitamin B6 was measured using high performance liquid chromatography (HPLC) by the method of Reynolds and Brain [24].

### 2.4 Follow-up

The men have been followed for mortality through flagging by the Health and Social Care Information Centre (HSCIC) with follow-up until 28th February 2012. The underlying cause of death coded from the death certificate was used. CVD deaths were defined as deaths from circulatory causes (ICD-9: 410–438 and 441 or ICD-10: I11, I20–I25, I42, I50–I51, I60–I69, I71 and I73), Non-CVD deaths were predominantly from cancers (50%) and from respiratory disease (22%). Accidental and violent deaths accounted for only 3% of non-CVD deaths.

### 2.5 Statistical analysis

1773 men had complete data on all relevant covariates and form the basis for the analysis. The majority of biomarkers showed skewed distributions and small numbers of men had results below a lower limit of detection for some biomarker assays. Biomarker results were therefore summarized using median and interquartile ranges and compared between subgroups using the Mann–Whitney U test. Biomarker distributions were divided into thirds by sample tertiles for further analysis.

Since many of the assays included in this report are only recently available, up to 30 duplicate serum or plasma samples blinded to each participating laboratory were included to assess repeatability. For two biomarkers assayed in Belfast pilot data indicated that the chosen ELISA kits offered poor reproducibility and another manufacturer's kits were used instead. Coefficients of variation, representing a combination of inter- and intra-run variation, were calculated. As assay variation was observed to increase in proportion to biomarker levels, pairs of biomarker readings were used to derive coefficients of variation which were then averaged across the pairs to give a single summary value. The end points used for this analysis were CVD death (n = 376) which included death from IHD (n = 265), stroke (n = 68), CHF (n = 7), aortic aneurysm (n = 27) and other circulatory causes (n = 9). Men with evidence of IHD at baseline or a history of stroke were classified as having prior CVD (n = 667) and these men were retained in the analysis with a covariate representing prior CVD included in the regression models. A competing risk Cox proportional hazards regression model [25] was used to estimate subhazard ratios (SHRs) for biomarker variables for each of CVD death and non-CVD death, with the other taken as a competing risk in the analysis. This approach models cumulative incidence functions rather than cause-specific hazard functions and, as has been pointed out in a cardiological context, is the more appropriate approach when the focus is on prognosis and medical decision making [26]. Tests for linear trend in the SHRs across the thirds of each biomarker were obtained together with tests for deviation from linearity across the thirds. An interaction term between prior CVD and each categorised biomarker was added to each model to test for evidence that the biomarker was more (or less) predictive in those with prior CVD than those without prior CVD. A time-dependent covariate test was used to investigate the subhazard proportionality assumption which specifies that there is no change in the subhazard ratio with time. A significant interaction between a categorised biomarker and time in the Cox model provided evidence of failure of the proportional subhazard assumption.

Initial analyses were adjusted only for age (not shown). Subsequent analyses added the following conventional risk factors as potential confounders: smoking (never/past/current), diabetes (yes/no), systolic blood pressure, total cholesterol, total triglycerides (logarithmically transformed), body mass index, history of cardiovascular disease (yes/no) and family history of CHD before 55 years of age (yes/no).

To reduce the risk of type 1 errors arising in the multiple testing, the 1% significance level was employed for all tests. Analyses were performed using SPSS version 20 (IBM Corp., Armonk, NY, USA) and Stata release 12 (StataCorp, College Station, TX, USA).

## 3 Results

The mean period of follow-up for the 1773 men was 15.4 years (range 0.1–22.3 years) with a total of 27,296 person-years.

Table 1 shows the median values (and interquartile range) in those men who were alive at the end of follow-up in 2012, in men who had died from CVD causes, and in those who had died from non-CVD causes. Significance values of P < 0.01 and P < 0.001 comparing median values in subjects who died of either CVD or of non-CVD causes with those alive at follow-up are also shown.

Thirteen biomarkers show significantly elevated levels in men who had subsequently died of CVD and three show significantly reduced levels. Fourteen show significant elevations in men who had died of non-CVD causes and three show significant reductions. Vitamin B6 shows significantly lower levels in those who died of both CVD and non-CVD causes, while VCAM and glutamate dehydrogenase show significantly lower levels only in men dying of CVD and cholesterol and RBP-4 show significantly lower levels only in men dying of non-CVD.

Table 2 shows the SHRs (with 95% confidence intervals) associated with a one-step increase across the thirds of distribution for each biomarker. Biomarkers are arranged in descending order of SHR for cardiovascular disease indicating the strength of their relationships with risk of death from CVD. SHRs which differ significantly from one (P < 0.01) are shown in bold type. The final column of Table 2 shows P values comparing these SHRs for CVD and non-CVD mortality.

Troponin and BNP each show a strong trend with risk of CVD death which is not shown with risk of death from non-CVD causes. Total cholesterol also shows an increased risk of CVD death while showing a strongly negative association with non-CVD mortality. Plasma viscosity, WCC, GDF-15, IL-6, and fibrinogen also show significant trends with risk of CVD death. Plasma viscosity, IL-6 and (GDF-15) were also significantly associated with risk of non-CVD death.

In addition to Troponin and BNP, C-reactive protein, alkaline phosphatase and GGT, all show significant positive relationships with non-CVD mortality (higher levels associated with increased risk) while total cholesterol, RBP-4 and vitamin B6 show significant negative associations (higher levels associated with reduced risk).

An interaction between prior CVD and each biomarker was included in the model but was found to be significant (P < 0.01) only for E-selectin in the CVD mortality analysis (Table 2, footnote c). Although Table 2 showed that overall E-selectin was not associated with a significant increase in CVD mortality risk, with a SHR of 1.15, 95% CI 0.99–1.32, it appeared that there was a significant excess risk (SHR 1.35, 95% CI 1.12–1.64) for the subgroup with prior CVD, but no significant excess risk in the subgroup without prior CVD (SHR 0.94, 95% CI 0.77–1.15).

**Fig. 1:**
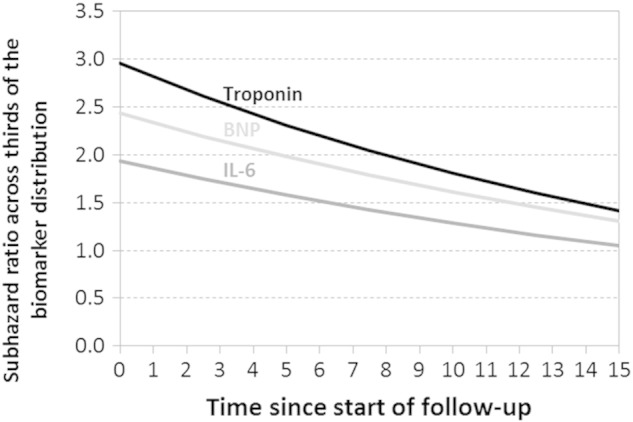
Estimated subhazard ratios for risk of cardiovascular death across thirds of the biomarker distributions of troponin, BNP, and IL-6 at different time points during follow-up.

A time dependent covariate analysis was used to check for evidence that the SHRs changed with the length of follow-up. Troponin, BNP and total cholesterol showed evidence of a stronger relationship with CVD mortality during the earlier years of follow-up (P < 0.01, Table 2, footnote a). These results are also illustrated in Fig. 1.

At time zero troponin shows an estimated SHR of 2.96 reducing to 1.42 by 15 years. IL-6 shows an initial SHR estimate of 1.93 reducing to 1.05 by 15 years. BNP has an estimated SHR of 2.44 at time zero falling to 1.31 by 15 years.

Cystatin-C and aspartate transaminase showed evidence of a non-linear relationship with CVD mortality (Table 2, footnote b). For both these biomarkers the lowest risk occurred in the middle third of the distribution of the biomarker. Our range of biomarkers showed evidence of neither time-dependency nor non-linearity in the relationship with non-CVD mortality.

## 4 Discussion

In this analysis we have examined the predictive value for both CVD and non-CVD mortality of a number of biomarkers from a range of possible pathological pathways after adjustment for conventional risk factors. Our list is not exhaustive but only a few biomarkers were omitted which show a consistent relationship with risk of CVD (e.g. HDL-cholesterol). We find that only the markers for cardiac necrosis and vascular function show a strong and specific relationship with CVD mortality, with the cell adhesion molecule VCAM and the protein PAPP-A each showing a stronger relationship with CVD than with non-CVD, but not achieving statistical significance.

Although many biomarkers have been investigated as potential markers of CVD, few have been found to usefully add to the clinical prediction of CVD [27]. However, recent large cohort studies have reported the role of troponins (particularly high-sensitivity troponins) [4,23,28] and the cardiac peptides BNP and NT-pro BNP [29] as important novel biomarkers for the prediction of CVD risk in the general middle-aged population. Both troponins and cardiac peptides add modestly to the risk of a CVD event in two European cohorts [4] and in a cohort from the USA [22] after adjustment for conventional risk factors. The period of follow-up was 10 years or less in these reports but longer in the present study (mean 15 years). Our results confirm these findings but also show that the risk is largest at the time of the initial examination declining substantially with the passage of time, but still showing a doubling of the risk per incremental third of the distribution by 15 years (Fig. 1).

Subjects with prior CVD were not excluded from our analysis. The importance of using competing risk approach when appropriate has been emphasised [26], but unfortunately our competing risk software did not accommodate stratification, which is our preferred approach to incorporating men with prior CVD in the regression model [2]. However, in the less appropriate standard Cox model analysis we found little difference between the biomarker hazard ratios obtained whether prior CVD was included in the model as a stratifying factor or as a covariate. So we chose instead to include prior CVD in the model as a covariate while checking that prior CVD showed no evidence of interaction with any biomarker that might indicate that it was more predictive in those with or in those without prior CVD. Only one such interaction was significant suggesting that E-selectin might be a predictor of CVD mortality only in those with a prior history of CVD; however some caution is advised in interpreting this finding in the light of multiple testing and the absence of a prior hypothesis.

Adhesion molecules such as E-selectin and VCAM are important in marking the interaction of leukocytes, platelets and vascular epithelium, which is an important pathway in the development of atherosclerosis and plaque formation [30]. In a follow-up study of patients with stable angina a number of adhesion molecules were measured at baseline, but only VCAM independently predicted risk of death from IHD adding to the risk prediction from the classical risk factors and C-reactive protein [12]. In the present study VCAM had a higher SHR for CVD than E-selectin, although neither attained statistical significance at the 1% level. PAPP-A is a metalloprotein secreted by coronary smooth muscle which, in a recent meta-analysis of 14 studies, was shown to be associated with higher mortality rates in prospective studies of patients with CHD [31]. In the present study PAPP-A is more specifically related to risk of CVD (although only at a more lenient 5% significance level) than non-CVD. Cystatin-C has been cited as an emerging risk marker for CVD [32], but is again only of borderline significance for this outcome in the present report.

Several of the inflammatory biomarkers (IL-6, GDF-15, C-RP) and the liver function tests alkaline phosphatase and GGT show a statistically significant association with risk for non-CVD death which exceeded the risk for CVD death, although the P values in the final column of Table 2 comparing the SHRs directly were not statistically significant.

The association of selected inflammatory markers has been noted in other reports in respect of total mortality (IL-6) [33] and for cause-specific mortality (cancers and respiratory disease) (C-reactive protein) [6,22], but the present report suggests that inflammatory markers may predict non-CVD at least as well as non-CVD. We examined deaths from cancers (n = 314) and respiratory deaths (n = 131) as separate causes of non-CVD mortality but, in a competing risk analysis, none of the biomarkers showed any tendency toward preferential association with either of these specific causes. Other non-CVD causes of death were too infrequent for separate analysis.

The liver enzymes aspartate and alanine transaminase have been associated with heart failure cross-sectionally and with the development of atrial fibrillation, an important cause of heart failure, longitudinally [34]. In the present study deaths from heart failure are probably under-represented (but are included under our heading of CVD) since ICD coding instructions recommend that the underlying cause of death should be used (which is IHD in more than 50% of our cases of heart failure that died). In the present report higher levels of these enzymes were not associated with increased risk of CVD. The enzyme aspartate transaminase also occurs in cardiac muscle and, before the introduction of troponins, was used in the diagnosis of acute myocardial infarction (when it was more commonly known as serum glutamic oxaloacetic transaminase (SGOT)). The liver enzyme GGT has been associated with increased risk of stroke (particularly with fatal and haemorrhagic stroke [35]) and with CVD and total mortality [20] the present study found no association with CVD but a significant association with non-CVD. The enzyme alkaline phosphatase occurs in both liver and bone and raised levels are associated with both CVD and non-CVD mortality. A recent meta-analysis of 19 cohort studies in which liver enzymes were measured suggested a 60% and a 38% increase in risk of death between extreme thirds of the distribution of GGT and alkaline phosphatase, respectively [36]. For aspartate transaminase there was a non-significant increase in risk (23%). For alanine transaminase there was a marked positive association in Asian populations (43% increase in all-cause mortality) but an 18% decrease in risk in North American populations.

RBP-4 and vitamin B6 show a significant negative association with risk of non-CVD (higher plasma levels appear to be associated with decreased risk). RBP-4 does not appear to have been investigated in large cohort studies but raised levels are associated with the development of insulin resistance in mice and with obesity and type 2 diabetes in human populations [37,38]. Low plasma levels of the active form of vitamin B6 have been consistently related to the development of colorectal cancer in five cohort studies [39] in contrast to findings from studies of dietary intake. Our findings support an association between non-CVD mortality and low levels of plasma vitamin B6, but lack the statistical power to differentiate the specific causes of death involved.

Total cholesterol shows a statistically significant relationship with risk of CVD although the association is weaker than six of the other significant biomarkers we studied (Table 2). However, it also shows a strong and statistically significant negative relationship with risk of non-CVD. This relationship has been shown in other cohort studies and has been attributed to a metabolic consequence of preclinical cancer and other non-CVD mortality [40–42]. In an analysis of cancer deaths (with non-cancer deaths regarded as a competing risk) removal of deaths in the first two years of the study only slightly weakens the association, with the adjusted SHR per step up the thirds of total cholesterol changing from 0.83, 95% CI 0.71–0.96 to 0.84, 95% CI 0.71–0.98; removal of deaths in the first five years of the study has a rather greater impact giving an adjusted SHR of 0.89, 95% CI 0.75–1.06.

This study provides a comprehensive and long-term assessment of a wide variety of biomarkers enabling us to test both specificity and time-dependent effects. However there are several important limitations that need to be considered. The study is not powered to test for associations with more specific causes of death and we have therefore limited our analysis to a CVD/non-CVD grouping. Our population-based sample consists only of men so our findings may not be applicable to women. Biomarkers were measured only at one time-point; repeat measures would be more informative and would assist in estimation of the effects of regression dilution bias. Assays were done in historical frozen samples without regard to possible storage effects, and an analysis based on fresh samples may show stronger associations. We have only analysed mortality but it is likely that these biomarkers also predict non-fatal events though the relative strength may not be the same for both fatal and non-fatal events.

## 5 Conclusions

Biomarkers from a range of pathogenic pathways showed significant associations with both CVD and non-CVD mortality. In our panel only five proved to be specific for CVD mortality (troponin, BNP, white cell count, fibrinogen and total cholesterol). We confirm the relevance of troponin and BNP as major biomarkers in the prediction of cardiovascular mortality and we found that the estimated SHRs were about twice the value at baseline of those at 15 years of follow-up. The inflammatory marker IL-6 also showed a weaker association with CVD mortality with length of follow-up, with no increased risk by 15 years. This was not the case for its association with non-CVD mortality.

Four out of the eight biomarkers in our panel that showed significant associations with CVD mortality were also significant for non-CVD mortality. An additional five biomarkers including C-RP, alkaline phosphatase and GGT had significant positive associations with non-CVD mortality while low levels of total cholesterol, RBP-4 and plasma vitamin B6 showed a significantly increased risk of non-CVD mortality.

## Author contributions

JY devised the study, obtained the funding, assisted in the data collection, compilation and analysis, and wrote the manuscript with CCP.

CCP advised on the statistical design of the study, compiled the data, conducted the data analysis and co-authored the manuscript.

SB participated in the study as Principal Investigator of *BiomarCaRE* and read and approved the final manuscript.

YB-S helped devise the project, assisted in the data collection, validated the clinical events, read and approved the final manuscript.

LH was responsible for collecting and compiling the outcome data and convening the validation committee, and he read and approved the final manuscript.

AB helped devise the project, participated in the data collection and validation committee, and read and approved the final manuscript.

GL helped devise the project, was responsible for the Glasgow biomarker assays, and read and approved the final manuscript.

TZ was responsible for the assays conducted within *BiomarCaRE*, and read and approved the final manuscript.

JG helped devise the project, and read and approved the final manuscript.

IY helped devise the project, had overall responsibility for the Belfast assays, and read and approved the final manuscript.

## Funding

The work was supported by the British Heart Foundation (PG/09/002/26056).

## Conflict of interest

The authors report no conflicts of interest.

## Figures and Tables

**Table 1:** Median (inter-quartile range) values of biomarkers in men alive at follow-up and in men dying from CVD and from non-CVD causes.

Biomarker	CV^a^	Alive			Dead — CVD cause			Dead — non-CVD cause		
n	Median	(Inter quartile range)	n	Median	(Inter quartile range)	n	Median	(Inter quartile range)
*Cardiac and vascular*										
Troponin (pg/mL)	13%	759	5.50	(4.50–6.90)	360	7.55⁎⁎	(5.73–11.08)	572	6.05⁎⁎	(4.70–7.90)
BNP (pg/mL)	13%	762	20.0	(12.8–31.6)	355	34.3⁎⁎	(19.3–66.0)	564	24.5⁎⁎	(15.4–40.9)

*Inflammatory*										
C-RP (mg/L)	4%	760	1.89	(1.07–3.74)	363	3.28⁎⁎	(1.55–6.57)	572	2.99⁎⁎	(1.39–5.91)
IL-6 (pg/mL)	17%	762	1.86	(1.28–2.70)	363	2.65⁎⁎	(1.73–3.83)	573	2.72⁎⁎	(1.89–4.10)
IL-6 receptor (ng/mL)	21%	760	37.9	(30.9–45.3)	363	39.9	(31.8–47.1)	575	38.6	(30.5–45.8)

*Lipids*										
Cholesterol (mmol/L)	1%	796	6.20	(5.60–6.90)	376	6.40	(5.60–7.18)	601	6.00⁎⁎	(5.30–6.80)
Triglyceride (mmol/L)	3%	796	1.60	(1.20–2.40)	376	1.70	(1.20–2.40)	601	1.50	(1.10–2.20)
Lp-a (mg/dL)	27%	755	7.70	(3.60–22.50)	363	8.60	(3.30–21.80)	571	7.80	(3.40–21.50)

*Adhesion molecules*										
VCAM (ng/mL)	8%	761	1259	(1131–1412)	362	1127⁎⁎	(1154–1530)	574	1299⁎⁎	(1170–1479)
E-selectin (ng/mL)	11%	762	25.2	(18.7–32.8)	362	28.2⁎⁎	(20.4–36.6)	574	27.0⁎⁎	(20.1–35.9)

*Liver function*										
Alkaline phosphatase (IU/L)	1%	793	80.0	(68.0–96.0)	376	89.5⁎⁎	(73.3–109.0)	593	90.0⁎⁎	(75.0–111.0)
Aspartate transaminase (IU/L)	6%	791	22.0	(18.0–26.0)	375	21.0	(17.0–25.0)	587	22.0	(18.0–27.0)
Alanine transaminase (IU/L)	5%	794	22.0	(17.0–29.0)	376	21.0	(16.0–27.0)	595	21.0	(17.0–28.0)
Gamma-GT (IU/L)	4%	796	28.5	(20.0–41.0)	376	29.0	(21.0–45.8)	601	31.0⁎	(22.0–47.5)
Glutamate dehydrogenase (IU/L)	25%	356	2.40	(1.30–3.60)	209	1.90⁎	(0.99–3.10)	289	2.10	(1.30–3.35)

*Acute phase*										
White cell count (× 10^9^/L)	4%	792	5.83	(5.02–6.87)	373	6.65⁎⁎	(5.42–7.85)	594	6.31⁎⁎	(5.24–7.50)
Viscosity (mPa/s)	1%	790	1.67	(1.62–1.73)	362	1.72⁎⁎	(1.66–1.78)	593	1.70⁎⁎	(1.62–1.78)
Fibrinogen (g/L)	5%	786	3.9	(3.5–4.5)	368	4.2⁎⁎	(3.7–4.9)	591	4.2⁎⁎	(3.6–4.7)
Ferritin (ng/mL)	8%	759	114	(67–198)	360	111	(63–185)	573	117	(61–206)

*Leukocyte activation*										
GDF-15 (pg/mL)	10%	762	618	(492–787)	363	803⁎⁎	(619–1051)	574	819⁎⁎	(633–1082)
PAPP-A (ng/mL)	11%	761	7.10	(5.25–9.19)	363	7.65⁎	(5.57–9.96)	573	7.20	(5.34–9.67)

*Renal function*										
Cystatin-C (mg/L)	2%	760	0.82	(0.74–0.90)	363	0.89⁎⁎	(0.79–0.98)	572	0.86⁎⁎	(0.79–0.98)
Creatinine (mg/dL)	1%	756	0.94	(0.86–1.03)	363	0.93	(0.83–1.05)	571	0.93	(0.85–1.03)

*Insulin resistance*										
Glucose (mmol/L)	6%	794	5.3	(4.9–5.7)	376	5.4⁎⁎	(5.0–6.1)	596	5.3⁎	(5.0–5.9)
Retinol binding protein 4 (mg/L)	18%	760	32.1	(26.9–38.9)	363	31.1	(25.4–37.8)	574	29.9⁎⁎	(24.6–36.2)
Fetuin-A (mg/L)	20%	760	258	(220–305)	363	259	(223–310)	573	259	(220–307)

*Others*										
Uric acid (mg/L)	4%	795	34.0	(29.0–39.0)	376	34.0	(29.0–40.0)	601	34.0	(29.0–39.0)
Vitamin B6 (nmol/L)	18%	763	42.8	(31.9–57.8)	353	36.2⁎⁎	(27.0–50.0)	569	36.1⁎⁎	(26.7–49.2)

a Coefficient of variation for reliability from blinded split samples.

⁎ P < 0.01, compared to the Alive group.

⁎⁎ P < 0.001, compared to the Alive group.

**Table 2:** Subhazard ratios (95% CI) for CVD and non-CVD mortality per third of each biomarker distribution.

Biomarker	CVD mortality subhazard ratio (95% CI)		Non-CVD mortality subhazard ratio (95% CI)		P (CVD versus non-CVD)
Troponin	**1.71**^a^	**(1.46–1.99)**⁎⁎	1.00^a^	(0.89–1.11)	< 0.001
BNP	**1.54**^a^	**(1.34–1.78)**⁎⁎	0.99	(0.89–1.10)	< 0.001
Plasma viscosity	**1.30**	**(1.13–1.50)**⁎⁎	**1.20**	**(1.08–1.34)**⁎⁎	0.49
White cell count	**1.28**	**(1.11–1.48)**⁎⁎	1.07	(0.96–1.19)	0.10
GDF-15	**1.25**	**(1.07–1.45)**⁎	**1.35**	**(1.20–1.53)**⁎⁎	0.53
IL-6	**1.24**^a^	**(1.08–1.43)**⁎	**1.38**	**(1.24–1.54)**⁎⁎	0.38
Total cholesterol	**1.20**	**(1.05–1.37)**⁎	**0.81**	**(0.72–0.90)**⁎⁎	< 0.001
Fibrinogen	**1.19**	**(1.05–1.36)**⁎	1.08	(0.98–1.20)	0.32
VCAM	1.19	(1.03–1.36)	1.01	(0.91–1.12)	0.10
Cystatin-C	1.19^b^	(1.02–1.38)	1.09	(0.98–1.21)	0.43
PAPP-A	1.16	(1.01–1.32)	0.97	(0.87–1.08)	0.07
E-selectin	1.15^c^	(0.99–1.32)	1.12	(1.01–1.24)	0.81
C-RP	1.12	(0.98–1.29)	**1.22**	**(1.09–1.36)**⁎⁎	0.42
Alkaline phosphatase	1.09	(0.96–1.24)	**1.27**	**(1.14–1.41)**⁎⁎	0.13
Glucose	1.09	(0.95–1.25)	1.13	(1.02–1.26)	0.70
Triglycerides	1.07	(0.93–1.22)	1.00	(0.90–1.11)	0.46
Uric acid	1.05	(0.91–1.21)	1.11	(0.99–1.23)	0.61
Fetuin-A	1.04	(0.91–1.18)	1.09	(0.98–1.21)	0.58
IL-6 receptor	1.02	(0.90–1.16)	1.00	(0.91–1.11)	0.85
Lp-a	0.99	(0.87–1.13)	1.01	(0.91–1.12)	0.82
GGT	0.98	(0.84–1.13)	**1.24**	**(1.11–1.38)**⁎⁎	0.02
RBP-4	0.95	(0.83–1.09)	**0.85**	**(0.77–0.95)**⁎	0.20
Vitamin B6	0.95	(0.83–1.09)	**0.83**	**(0.75–0.93)**⁎	0.10
Ferritin	0.93	(0.81–1.06)	1.05	(0.95–1.17)	0.13
Alanine transaminase	0.92	(0.81–1.06)	1.07	(0.96–1.19)	0.09
Creatinine	0.90	(0.80–1.03)	1.04	(0.94–1.14)	0.08
Aspartate transaminase	0.89^b^	(0.77–1.02)	1.10	(0.99–1.21)	0.01
Glutamate dehydrogenase	0.84	(0.70–1.01)	1.06	(0.92–1.23)	0.03

a Evidence of departure from proportional subhazards assumption (P < 0.01).

b Evidence of non-linearity in the relationship between biomarker and mortality (P < 0.01).

c Evidence of biomarker interaction with prior CVD (P < 0.01).

⁎ P < 0.01.

⁎⁎ P < 0.001.
